# Optimizing the use of cesarean section in Argentina: design and methodology of a formative research for the development of interventions

**DOI:** 10.1186/s12978-021-01080-4

**Published:** 2021-01-26

**Authors:** Silvina Ramos, Mariana Romero, Carla Perrotta, Yanina Sguassero, Cecilia Straw, Celina Gialdini, Natalia Righetti, Ana P. Betran

**Affiliations:** 1 CEDES: Centro de Estudios de Estado y Sociedad, Buenos Aires, Argentina; 2 Consejo Nacional de Investigaciones Científicas y Técnicas, CONICET/CEDES: Centro de Estudios de Estado Y Sociedad, Buenos Aires, Argentina; 3 School of Public Health, University College Dublin, National University of Ireland, Dublin, Ireland; 4 CREP: Centro Rosarino de Estudios Perinatales, Rosario, Argentina; 5 Facultad de Ciencias Sociales, Universidad de Buenos Aires/CEDES: Centro de Estudios de Estado Y Sociedad, Buenos Aires, Argentina; 6 Department of Reproductive Health and Research, UNDP, UNFPA, UNICEF, WHO, World Bank Special Programme of Research, Development and Research Training in Human Reproduction, World Health Organization, Geneva, Switzerland

**Keywords:** Cesarean section, Public hospitals, Argentina, Qualitative research

## Abstract

**Background:**

While cesarean section is an essential life-saving strategy for women and newborns, its current overuse constitutes a global problem. The aim of this formative research is to collect information from hospitals, health professionals and women regarding the use of cesarean section in Argentina. This article describes the methodology of the study, the characteristics of the hospitals and the profile of the participants.

**Methods:**

This formative research is a mixed-method study that will be conducted in seven provinces of Argentina. The eligibility criteria for the hospitals are (a) use of the Perinatal Information System, (b) cesarean section rate higher than 27% in 2016, (c) ≥ 1000 deliveries per year. Quantitative and qualitative research techniques will be used for data collection and analysis. The main inquiry points are the determining factors for the use of cesarean section, the potential interventions to optimize the use of cesarean section and, in the case of women, their preferred type of delivery.

**Discussion:**

It is expected that the findings will provide a situation diagnosis to help a context-sensitive implementation of the interventions recommended by the World Health Organization to optimize cesarean section use.

*Trial registration* IS002316

**Plain English Summary:**

Cesarean section is an essential medical tool for mothers and their children, but nowadays its overuse is a problem worldwide. Our purpose is to get information from hospitals, health professionals and women about how cesarean section is used in Argentina. In this protocol we describe how we will carry out the study and the characteristics of the hospitals and participants. We will implement this study in seven provinces of Argentina, in hospitals that have more than 1,000 births each year, had a cesarean section rate higher than 27% in 2016 and use the Perinatal Information System. We will gather information using forms, surveys and interviews. We want to identify the factors that decide the use of a cesarean section, the potential interventions that can improve the use of cesarean section and, in the case of women, the type of delivery they prefer. We expect that this study will give us a diagnosis of how cesarean section is used in Argentina, and that this will help to apply the interventions that the World Health Organization recommends to optimize the use of cesarean section in our specific context.

## Resumen

**Introducción:**

Aun cuando la cesárea es una intervención que puede ser esencial para salvar la vida de una mujer y su hijo, el crecimiento excesivo de su uso  es un problema global. El propósito de esta investigación formativa es recolectar información sobre las instituciones, profesionales de la salud y mujeres acerca del uso de la cesárea en la Argentina. Este artículo describe la metodología del estudio, las características de los hospitales y el perfil de los participantes.

**Metodología:**

Esta investigación formativa usa un diseño mixto aplicado en siete provincias del país. Los criterios de elegibilidad para los hospitales son: (a) uso del Sistema Informático Perinatal, (b) tasa de cesáreas mayor al 27% en 2016, y (c) ≥ 1000 partos por año. Se usarán técnicas cualitativas y cuantitativas para la recolección de datos y el análisis. Los principales temas a indagar son los determinantes del uso de la cesárea, las intervenciones para optimizar su uso y, en el caso de las mujeres, sus preferencias sobre el modo de parto.

**Discusión:**

Se espera que los resultados den lugar a un diagnóstico de situación que permita una implementación de las intervenciones propuesas por la OMS para optimizar el uso de la cesárea más ajustada al contexto.

*Registro del estudio* IS002316

## Background

During the past century, childbirth went from being a domestic, familiar and community matter to a professional medical act. Changes in how child delivery is perceived and in the use of interventions and new technologies resulted in significant health improvements and, at the same time, took the experience of childbirth out of the family setting and into hospital facilities [1]. One of the interventions that resulted from the improvements introduced by the change in the child delivery care model was cesarean section. Since then, it has become a fundamental life-saving strategy for women and newborns, and it is also one of the emergency OB strategies that the World Health Organization (WHO) considers essential [2].

Cesarean section is a surgery that is used to solve or prevent certain complications that occur during pregnancy or childbirth and to reduce health risks for women and newborns. However, like any surgical procedure, cesarean section presents some risks and its use implies greater costs for health systems [3–5]. The inappropriate use of this intervention is a cause of concern given the fact that, as in highly vulnerable social contexts some women do not have access to a cesarean section and in others it is unnecessarily used, it increases inequalities in the access to and use of health interventions in the population.

Though cesarean section rates higher than 15–20% have not shown to have a positive impact on perinatal results [6, 7], the sustained increase in cesarean section rates above those values is a global issue [7]. It is estimated that there was a 3.7% increase in the global average rate of cesarean section use during 2000–2015 and several countries have reported 40–50% cesarean section rates in their populations [7]. Despite the fact that cesarean section has specific clinical indications, there is usually no homogeneous criteria in the use of this intervention and the previously mentioned increase would be the result of a greater use of cesarean section without any clinical indication, mainly in high and middle income countries [6]. Financial incentives, availability of resources in the institutions, health services beliefs and skills, organizational culture aspects, women's characteristics and preferences, among other factors, affect OB practice and may determine the use of cesarean section [8–12].

Evidence has shown that when isolated interventions are used to address the situations and processes that lead to an increase in the use of cesarean section in clinical practice, the results obtained as to a reduction in its use are less effective [13]. Furthermore, multiple component interventions that include all the stakeholders involved (women, health workers and health systems) have shown to present a higher probability of reducing the incidence of unnecessary cesarean sections [13–15]. However, the implementation of this kind of interventions is very challenging given the diversity in health care organization, health services practices, the many barriers that must be overcome in order to introduce and sustain the changes promoted by these interventions and also because these interventions demand a change in individual behaviors and affect the organizational culture of services and institutions [13, 16]. This is why the WHO recommends that, before implementing any intervention, a formative research be carried out to identify and define why there is an increase in rates in a specific environment, why this is locally relevant, which the determining factors are for this phenomenon and what women and health professionals think within the framework of certain cultural rules pertaining to the specific contexts [13].

Both South America and the Caribbean are regions with some of the highest cesarean section rates in the world, and both show a sharp acceleration in their increase [7]. Argentina is no stranger to this situation: the national average rate in public health subsector institutions increased by 23% between 2010 and 2017, to reach 34.7% ranging between 28.4% and 57.7% in 2017 [17]. This heterogeneous increase would indicate that the use of cesarean section might be affected by reasons that are not necessarily clinical. In addition, according to a previous research, women in Argentina prefer vaginal delivery [9],which demands a better understanding of the role of women’s preferences as well as the dynamics of the decision-making process in maternal and infant health, and, more specifically, of the organizational cultures in which decisions as regards performing a cesarean section are made. It is also necessary to understand the relationship that health professionals and services have with women in the hospital obstetric care setting. In this context, formative research has great potential.

Formative research has shown to be key in the design, development and execution of health interventions as it makes it possible to identify the characteristics of the stakeholders involved and to adjust the interventions to the peculiarities of the socio-cultural and institutional contexts [18, 19]. This type of research informs about the stakeholders’ beliefs, values, attitudes, knowledge and behavior in relation to a particular problem and its context. Formative research also provides evidence as to why some interventions are effective and others are not, it makes it possible to understand which factors in the health care process contribute to making an intervention implementation satisfactory, and it identifies the culturally appropriate interventions to be tested [16].

The purpose of our formative research is to collect information from hospitals, health professionals and women as regards the conditions, use, preferences, and potential barriers and facilitators to inform the design and the implementation of non-clinical interventions aimed at optimizing the use of cesarean section in public maternity hospitals in Argentina. This article describes the design, research methodology, and profile of study samples and it is part of a series of publications.

## Methods

This study uses a mixed-method design that combines quantitative and qualitative data collection and analysis techniques. The quantitative techniques include an institutional form that collects the characteristics of the participating hospitals, an online structured survey questionnaire to be applied to the health services and an in-person interview to be applied to the women who gave birth at those hospitals. The qualitative aspect of the study includes a subgroup of hospitals and it is based on semi-structured interviews applied to key informants. Finally, after finishing the data collection stage, a working meeting will be held with the heads of services and heads of midwives in order to validate the preliminary results and explore some hypothesis that may facilitate the interpretation of findings.

Eligibility criteria for public hospitals across Argentina are the following: a) use of the national perinatal information system (SIP-gestión); b) cesarean section rate higher than 27% in 2016; c) more than 1000 deliveries per year [17].

Out of the 88 potentially eligible institutions, 24 were non-randomly selected from the six country regions. 19 institutions agreed to participate, out of which five were selected, one from each country region, except for the Patagonian region where the institution declined to participate and to apply the in-person interview to women and the semi-structured interview to key informants.

The project was approved by the Independent Ethics Committee of Centro Rosarino de Estudios Perinatales and by the provincial Ethics Committees and/or the Teaching and Research Committees at each of the selected hospitals pursuant to the requirements in each jurisdiction. It was also approved by two Ethics Committees of WHO, the Research Project Review Panel of the UNDP/UNFPA/UNICEF/WHO/World Bank Special Programme of Research, Development and Research Training in Human Reproduction (HRP) at the Department of Sexual and Reproductive Health and Research of WHO, and the WHO Research Ethics Review Committee, Geneva, Switzerland. In Argentina, the research protocol was registered in the RENIS database (number IS002316).

The data collection tools are designed to collect information regarding three dimensions: determining factors for the use of cesarean section, interventions that may optimize the use of cesarean section and, in the case of women, their preferences as to the type of delivery (vaginal vs. cesarean section) and the reasons for such preference. Four tools have been designed: (1) institutional form; (2) health professionals’ form to be completed online; (3) women's form to be completed during an in-person interview and (4) hospital key informants’ form to be completed during an in-person interview. Every tool, explained in detail below, will be tested before starting the fieldwork.

### Data collection and tools

The institutional form collects information regarding the structural and organizational culture aspects: type and characteristics of the institution (number of live births in 2017, number of cesarean sections, training for general practice and obstetric residents, availability of ORs, labor and delivery units, newborn intensive care and obstetric beds); production (number of deliveries and cesarean sections, human resources, availability of supplementary services, including motherhood comprehensive preparedness programs); availability of pain management interventions during labor; and conditions for accompaniment during labor and delivery. It is completed by an institutional focal point especially chosen for the research in coordination with the head of service.

The purpose of the health professionals’ online survey is to obtain their inputs and personal opinion about the three dimensions as well as about a set of institutional and clinical practices related to the use of cesarean section. This survey is sent to the whole eligible population, which includes all the (permanent staff and shift personnel) specialized physicians and obstetricians and residents at the obstetrics & gynecology and obstetrics services at the selected hospitals. The survey includes a participation consent (compulsory answer) and 41 questions, 36 of them with pre-established options and 5 open questions. The questions address the following variables: (a) role and healthcare work, including the number of deliveries/cesarean sections performed in a week, (b) cesarean section deliveries at the hospital, (c) agreement with certain determining factors for the use of cesarean section in relation to the institution, health professionals and pregnant women, (d) interventions to optimize the use of cesarean section at the hospital, and (e) use of monitoring cycles and improvement in quality of care. This tool has been designed with a five-option Likert scale with the options “strongly agree” and “strongly disagree” as extreme answering options. For the interventions, the questions address usefulness and feasibility. Up to four reminders will be sent for the survey, one every seven days until the four-week time frame of the field work is completed. To reinforce the survey confidentiality, the respondents’ gender will not be recorded.

The women's interview is applied to postpartum women eight hours after delivery. Inclusion criteria are the following: (a) having had one delivery (vaginal or cesarean section) at the participating hospital, (b) ≥ 15 years old, (c) the newborn had not required hospitalization in Neonatal Intensive Care Unit, and  (d) obtaining the informed consent. Based on the average number of childbirths per institution, a minimum total number of 450 completed interviews is estimated. For this purpose, the adopted strategy is the selection of a consecutive sample in which one woman out of four who has a vaginal or cesarean section delivery is taken every day of the week until the 3 months of the survey are completed or 130 cases in each hospital are included (whichever criteria is met first). This tool consists of a structured survey questionnaire with 18 closed questions and 9 open ones. The variables covered by the survey questions are the following: (a) reproductive history, (b) opinions and preferences as regards vaginal and cesarean section delivery, (c) labor and delivery process (including accompaniment), (d) reasons to perform a cesarean section, vaginal and cesarean section delivery advantages and disadvantages, and (e) women's preference in general. The survey is applied by a health care provider specially trained for this task (social worker, psychologist or nurse) who is not a member of the obstetrics and gynecology service to prevent any bias and to preserve confidentiality.

The interviews to key informants are applied to 25 health professionals (five per hospital). In order to guarantee an heterogeneity in responsibilities and roles in obstetrics care, heads of services, residents and physicians specialized in obstetrics & gynecology, midwives and midwifery residents will be interviewed. A 16 open questions guide is used to inquire about the following variables: (a) factors that affect the decision as to which type of delivery to provide; (b) reasons involved in the use of cesarean section (professional skills to handle a complex or dystocial delivery, delivery after a cesarean section, problems related to pain management, characteristics of the population to which they provide attention, legal aspects, etc.); (c) professional opinion regarding the use of cesarean section; (d) analysis of the obstetrics service indicators; (e) experiences with changes in such services; (f) professional predisposition to change; and (g) usefulness and feasibility of the interventions to optimize the use of cesarean section (ongoing training in obstetric emergencies, strategies to improve adherence to clinical guidelines and protocols, training in the performance of instrumental vaginal deliveries and management of other high risk situations using high fidelity simulations, application of Robson classification, organization of human resources, financial or other incentives, interventions aimed at women). Interviews are carried out by especially trained personnel external to the services, in-person or virtually at the convenience of the interviewed participants.

### Data analysis

For the quantitative analysis, the level of agreement with the statements will be described. The variance in the answers of health care professionals will be assessed to see whether they are more similar to the answers of other health professionals in the same hospital or to similar professions across different hospitals. For the women’s survey, data will be described as mean ± standard deviation (mean ± SD) or median (range) for continuous variables and percentages for categorical variables. Chi-square test will be used for categorical variables as group comparison. Preference for mode of delivery will be analyzed using univariate and multivariate analysis adjusting for maternal age, delivery mode on the index pregnancy, parity, history of miscarriage, and delivery hospital. Unadjusted Odds Ratio (OR) and adjusted OR and 95% Confidence Interval (CI) will be used. All statistical tests of hypotheses will be two sided and criterion for statistical significance will be set to α = 0.05. Statistical analyses will be carried out with Stata version 15.

Qualitative data will be thematically analyzed [20]. Also, as different actors interact in the processes under study, focus is placed on understanding, by means of detailed descriptions, the different points of view according to the role or experience throughout the process, captured in their own words, and in the specific contexts in which they take place [21].

Finally, a validation meeting will be held to present the results and facilitate their discussion by the research team members. This will be a whole working day in-person meeting.

## Discussion

The need to design evidence-informed and context-sensible interventions which may be sustained in time is imperative for public policies aimed at neonatal care [22, 23]. In 2018, the World Health Organization published a guideline on the use of non-clinical interventions to reduce the number of unnecessary cesarean sections [13]. In that guideline, some gaps in the available knowledge were identified in relation to the uncertainty as to the effects of the interventions so as to inform the development of national policies and protocols, as well as in relation to the applicability of the evidence in diverse cultural and institutional contexts. The guideline also recommends four non-clinical interventions in order to reduce unnecessary cesarean sections: interventions on women, on service providers and on institutions and health systems. Finally, and fundamentally, it is concluded that formative research is necessary to guide the implementation of strategies aimed at reducing or optimizing the use of cesarean sections, as it makes it possible to identify the determining factors in the use of cesarean section and other factors that may affect the implementation of interventions at a local level [13, 14].

Following these recommendations, the WHO developed a generic protocol to guide the design of a formative research prior to the performance of interventions aimed at optimizing the use of cesarean section at a local level, on the understanding that this type of research might be useful to understand the problems, challenges and people affected by this problem as well as its causes [16]. This article presents the design and methodology of a formative research that falls into the same line of work prioritized by WHO and that agree with its protocol. Our research proposal is thus developed at the forefront of multi-component studies implementation which include all the parties that have an interest in optimizing the use of cesarean section throughout the world. Hospitals with a large volume of patients and most of them with a good infrastructure and resources given their designation as secondary or tertiary level maternity hospitals will be included, according to the perinatal categorization system.

There are important challenges in the planning and implementation of this research, more specifically those related to methodological aspects. For instance, challenges were faced at obtaining the consent from the institutions to participate in the research, given its innovative approach which includes collecting the personnel and women's opinions. Some challenges that may appear in the next phases of the study are, on the one hand, the application of the self-administered online survey without any economic incentive and, on the other hand, the result validation meeting, which will entail an interaction rarely used at two levels: between decision makers and researchers and between result presentation and collective validation. Regarding the latter, some facilitating elements may be the academic recognition of the participating institutions, working relationships prior to the research and inclusion in the study of a referent at each institution to promote consent.

From an ethical and equity point of view, every woman has the right to a good quality and respectful treatment and care during pregnancy and delivery and this includes the right to the appropriate use of cesarean section when necessary. Health services shall be organized and structured in such a way that these rights be promoted and protected. The purpose of our research is to improve the understanding of the preferences and their “why” (both in women as well and in health providers), the obstacles and the facilitating elements. With this purpose, this research will act as a catalyst for the most efficient and equitable implementation of interventions to improve care for women.

The results of this research are expected to enable a situation diagnosis in such a way that will make it possible to adapt the recommended interventions to optimize the use of cesarean section. Our findings will contribute to the design of strategies for a more context-sensible implementation. The possibility of having information from the point of view of health professionals and women will increase the chances of acceptance and sustainability of those interventions. More specifically, the results of this research will contribute to the preliminary qualitative work in which the design and implementation of the QUALI-DEC (Adequate use of cesarean section by means of quality decision making by women and health professionals “QUALI-DEC”) study intervention will be based [24]. QUALI-DEC is a strategy with four active ingredients: (1) opinion leaders to improve physicians' compliance with evidence-based practices; (2) auditing of the cesarean section indications to help providers identify potentially unnecessary cesarean sections; (3) tool to help women make an informed decision as regards the type of delivery; and (4) Companion chosen by the woman during delivery to support women during vaginal delivery. This research will be implemented in four countries, including Argentina.

## Data Availability

The data will be stored on CEDES’ server, encrypted. CEDES will be the guardian of the dataset. Data will be anonymized. Our data collection forms do not include any variable that could reveal the identity of the participants.

## Abbreviations

RENIS: Registro Nacional de Investigaciones en Salud (National Registry of Health Research)
SIP: Sistema de Información Perinatal (National Perinatal Information System)
QUALI-DEC: QUALIty DECision-making by women and providers
HRP: The UNDP/UNFPA/UNICEF/WHO/World Bank Special Programme of Research, Development and Research Training in Human Reproduction
UNDP: United Nations Development Programme
UNFPA: United Nations Fund for Population Activities
UNICEF: United Nations International Children's Emergency Fund
WHO: World Health Organization

## References

[1] Drife J (2002). The start of life: a history of obstetrics. Postgrad Med J.

[2] World Health Organization, UNFPA, UNICEF and Mailman School of Public Health. Averting Maternal Death and Disability (AMDD). Monitoring emergency obstetric care. A Handbook. Geneve: Word Health Organization; 2009. https://www.who.int/reproductivehealth/publications/monitoring/9789241547734/en/

[3] Keag OE, Norman JE, Stock SJ (2018). Long-term risks and benefits associated with cesarean delivery for mother, baby, and subsequent pregnancies: Systematic review and meta-analysis. PLoS Med.

[4] Cook JR, Knight M, Dhanjal MK (2013). Multiple repeat caesarean section in the UK: incidence and consequences to mother and child: A national, prospective cohort study–authors' reply. BJOG.

[5] Timor-Tritsch IE, Monteagudo A (2012). Unforeseen consequences of the increasing rate of cesarean deliveries: early placenta accreta and cesarean scar pregnancy. A review AJOG.

[6] Boerma T, Ronsmans C, Melesse DY, Barros AJD, Barros FC, Juan L (2018). Global epidemiology of use of and disparities in caesarean sections. Lancet.

[7] Betran AP, Ye J, Moller AB, Zhang J, Gülmezoglu AM, Torloni MR (2016). The Increasing Trend in Caesarean Section Rates: Global, Regional and National Estimates: 1990–2014. PLoS ONE.

[8] Kingdon C, Baker L, Lavender T (2006). Systematic Review of Nulliparous Women’s Views of Planned Cesarean Birth: The Missing Component in the Debate about a Term Cephalic Trial. Birth.

[9] Mazzoni A, Althabe F, Liu NH, Bonotti AM, Gibbons L, Sánchez AJ, Bélizan JM (2011). Women’s preference for caesarean section: a systematic review and meta-analysis of observational studies. BJOG.

[10] Litorp H, Mgaya A, Kidanto HL, Johnsdotter S, Essén B (2015). “What about the mother?” Women’s and caregivers’ perspectives on caesarean birth in a low-resource setting with rising caesarean section rates. Midwifery.

[11] Aminu M, Utz B, Halim A, van den Broek N (2014). Reasons for performing a caesarean section in public hospitals in rural Bangladesh. BMC Pregnancy Childbirth.

[12] Bohren MA, Opiyo N, Kingdon C, Downe S, Betrán AP (2019). Optimising the use of caesarean section: a generic formative research protocol for implementation preparation. Reprod Health.

[13] World Health Organization. WHO recommendations non-clinical interventions to reduce unnecessary caesarean sections. Geneve: World Health Organization; 2018. https://www.who.int/reproductivehealth/publications/non-clinical-interventions-to-reduce-cs/en/30398818

[14] Chen I, Opiyo N, Tavender E, Mortazhejri S, Rader T, Petkovic J (2018). Non-clinical interventions for reducing unnecessary caesarean section. Cochrane Database Syst Rev.

[15] Scioscia M, Vimercati A, Cito L, Chironna E, Scattarella D, Selvaggi LE (2008). Social determinants of the increasing caesarean section rate in Italy. Minerva Ginecol.

[16] Bohren MA, Opiyo N, Kingdon C, Downe S, Betrán AP (2019). Optimising the use of caesarean section: a generic formative research protocol for implementation preparation. Reproductive Health.

[17] Ministerio de Salud y Desarrollo Social. Sistema Informático Perinatal para la Gestión (SIP-G) Indicadores básicos 2017. República Argentina Buenos Aires: 2018. http://www.msal.gob.ar/images/stories/bes/graficos/0000001376cnt-anuario-sip-2017.pdf

[18] Gittelsohn J, Steckler A, Johnson C, Pratt C, Grieser M, Pickrel J, et al. Formative research in school and community-based health programs and studies: "state of the art" and the TAAG approach. Health Educ Behav. 2006;33(1):25–39. https://www.ncbi.nlm.nih.gov/pmc/articles/PMC2475675/10.1177/1090198105282412 PMC2475675 16397157

[19] Higgins DL, O'Reilly K, Tashima N, Crain C, Beeker C, Goldbaum G, Elifson CS, Galavotti C, Guenther-Grey C. Using formative research to lay the foundation for community level HIV prevention efforts: an example from the AIDS Community Demonstration Projects. Public Health Rep. 1996;111 Suppl 1(Suppl 1):28–35. PMC1382040 8862154

[20] Braun V, Clarke V (2006). Using thematic analysis in psychology. Qualitative Research in Psychology.

[21] Patton MQ (2014). Qualitative research and evaluation methods.

[22] Betrán AP, Temmerman M, Kingdon C, Mohiddin A, Opiyo N, Torloni MR (2018). Interventions to reduce unnecessary caesarean sections in healthy women and babies. Lancet.

[23] Opiyo N, Kingdon C, Oladapo OT, Souza JP, Vogel JP, Bonet M (2020). Non-clinical interventions to reduce unnecessary caesarean sections: WHO recommendations. Bull World Health Organ.

[24] isrctn.com. 2020 [actualizado 1 abril 2020]. http://www.isrctn.com/ISRCTN67214403

